# Stress Management in Cyst-Forming Free-Living Protists: Programmed Cell Death and/or Encystment

**DOI:** 10.1155/2015/437534

**Published:** 2015-01-12

**Authors:** Naveed Ahmed Khan, Junaid Iqbal, Ruqaiyyah Siddiqui

**Affiliations:** Department of Biological and Biomedical Sciences, Aga Khan University, Karachi 74800, Pakistan

## Abstract

In the face of harsh conditions and given a choice, a cell may (i) undergo programmed cell death, (ii) transform into a cancer cell, or (iii) enclose itself into a cyst form. In metazoans, the available evidence suggests that cellular machinery exists only to execute or avoid programmed cell death, while the ability to form a cyst was either lost or never developed. For cyst-forming free-living protists, here we pose the question whether the ability to encyst was gained at the expense of the programmed cell death or both functions coexist to counter unfavorable environmental conditions with mutually exclusive phenotypes.

## 1. Introduction 

Cell death is not a “dead subject.” It has intrigued the scientific community for centuries and rightly so. Being able to induce or prevent cell death can give us an advantage over other species. There has been tremendous progress in our understanding of the cell death. Unless forced to die (referred to as “necrosis”), a cell can determine its own fate in the face of harsh conditions. In this context, cell death is not an action but a reaction. But why would a reaction be to kill itself? For higher eukaryotes, it is well established that a cell will commit suicide in response to callous conditions or genomic instability for the greater benefit/survival of the organism. But, for a unicellular organism that is programmed for survival, it seems more logical to endure unfavorable conditions than to exterminate itself. It is presumed that such organisms should only consider “suicide” if they are unable to endure harsh conditions. Here we debate programmed cell death in the context of cyst-forming and non-cyst-forming protists that are important to human health.

## 2. Metazoan Cellular Stress Management in a Programmed Manner

For metazoans, the programmed cell death function is critical to eliminate unnecessary or unhealthy cells, following cellular stress. It is initiated by transduction of stress signals originating from extracellular (extrinsic) or intracellular (intrinsic) sources. Extrinsic signals may include ligands such as Fas, tumor necrosis factor that binds to cellular surface receptors leading to the activation of executioner caspases. In the intrinsic pathway, mitochondrial dysregulation is a prominent feature that triggers the release of proapoptotic proteins and cytochrome* c*, leading to the activation of executioner caspases that inflict death [1]. Metazoan cells can evade signal-induced programmed cell death function by accumulating a series of genetic alterations and becoming a cancer cell.

## 3. Programmed Cell Death in Parasitic Protists That Are Unable to Form Environmentally Viable Cysts, in Response to Stress 

This group of parasites includes* Plasmodium *spp.,* Trypanosoma* spp.,* Leishmania* spp.,* Babesia* spp., and* Trichomonas vaginalis*, which produce serious human infections [2–4]. Although* Plasmodium* can make themselves oocyst, a survival form against harsh environments, they are not viable in the environment and are for simplicity described as non-cyst-forming parasitic protists. In general, the inability of this group of organisms to form cysts means that they cannot survive in the environment. They must reside inside a host at all times, be that a vertebrate or an invertebrate host. It is unclear whether this group of organisms lost the ability to form cysts as a consequence of a long history of coevolution with its host or never developed it. The apparent net result is that the fate of the parasite is determined by the host viability; that is, the death of the host warrants and possibly induces parasite death. This group of organisms is shown to undergo cell death, in response to harsh conditions such as reactive oxygen species, nitric oxide, hydrogen peroxide, increased temperatures, and drugs (reviewed in [5, 6]). For example, studies have shown that under stress* Leishmania* spp. exhibit DNA fragmentation, expression of caspase-like peptidases, release of cytochrome* c*, phosphatidylserine exposure, and translocation of endonuclease G, which are the hallmarks of programmed cell death [7]. Similarly, stress-induced cell death is reported in* Plasmodium* spp. [8–10],* Trypanosoma* spp. [11, 12], and* Trichomonas vaginalis* [13] and linked to several markers that are characteristics of mammalian programmed cell death. Alternatively, it is possible that the cell death is induced or inhibited by the parasite as a means to achieve a stable niche so as not to kill the host [5, 14, 15].

## 4. Stress Management in Cyst-Forming Pathogenic Free-Living Protists

Cyst-forming free-living protists (examples are* Acanthamoeba *spp.,* Balamuthia mandrillaris*, and* Naegleria fowleri*) can propagate independently in the environment, without the need of a host [2]. They do not seek humans or other hosts to infect and proliferate but produce disease upon accidental encounter with humans. This group of organisms thrives in their natural habitat as free-living organisms. Under stressful conditions such as starvation and extremes in temperatures, pH, osmolarity, irradiation, and drugs, they rapidly transform from a metabolically active trophozoite stage into a dormant cyst form (metabolically inactive or of minimal metabolic activity). As long as harsh environmental conditions persist, they remain encysted. The cyst form can remain viable for more than 20 years without losing their pathogenicity [16, 17]. The return of favourable conditions is stimuli for activating metabolic pathways accompanied with excystment (i.e., reversion into the trophozoite stage) leading to reproduction of their species. It is interesting that some of the aforementioned stress signals are life-threatening for non-cyst-forming parasitic protists (possibly via programmed cell death) but induce encystment in cyst-forming free-living protists. Consistent with programmed cell death, encystment is a metabolically active, energy-dependent process requiring ATP for signaling from the cytoplasm to the nucleus of the cell and can take up to several hours. It is puzzling to understand whether encystment and programmed cell death coexist in this group. If so, what are the mechanics of coexistence? In response to harsh conditions, how does a cyst-forming free-living protist decide to either encyst or execute programmed cell death? What insults/injuries acting as triggers lead the organism to determine its own fate, that is, to become dormant but viable or commit suicide? Recent studies have shown that bacterial-treated* Acanthamoeba* (representative cyst-forming free-living protist) exhibit features indicative of mammalian programmed cell death [18, 19], albeit clear evidence of programmed cell death is lacking. These findings suggest that the molecular machinery required for the initiation and execution of programmed cell death may exist in* Acanthamoeba* [18, 19], although biochemical features (metazoan counterparts) and homologs of proapoptotic proteins that orchestrate programmed cell death in cyst-forming free-living protists are yet to be identified. Likewise, the regulation of programmed cell death at the transcriptional, translational, and posttranslational level is not known. Based on the limited available evidence, it is reasonable to suggest that both energy-dependent functions, that is, programmed cell death and encystment, coexist in this group of organisms. But can both be activated simultaneously? Do both complement or counter each other for the benefit of the organism? What are the stress triggers? How are they differentiated? What are the cellular receptors and the underlying mechanisms of activation? How are these signals mediated intracellularly? What are the potential executioner molecules and/or analogues of metazoan programmed cell death? Previous studies using* Acanthamoeba* as a model organism [20, 21] showed that nutrient deprivation does not produce 100% encystment, suggesting that only a subpopulation of amoebae cells at a given stage in their life cycle are able to encyst. They further showed that only cells that had completed 80% of the cell cycle are able to respond to encystment trigger, that is, nutrient deprivation. The question arises, what happens to others who are unable to encyst but face harsh conditions? Byers et al. [22] suggested that competence for encystment is limited to a portion of the cell cycle of* Acanthamoeba*. Is it possible that, under harsh conditions, cells competent to encyst (within 80% of the cell cycle) undergo encystment, while others (very young and very old) execute programmed cell death? From a community point of view, it makes sense to have only the fit ones (that can endure harsh conditions) to remain viable. But how are others (young and old ones) sacrificed? Is it self-destruction via programmed cell death or induced programmed cell death by the remaining competent population through mechanisms such as quorum sensing? This could be tested by treating* Acanthamoeba* trophozoites with conditioned medium of encysting cells to see whether cells undergoing encystment produce any quorum sensing molecule(s), which produce any effect on nonencysting cells. In addition, the existence of programmed cell death can be tested by exposing asynchronous cultures to nutrient-deprived conditions and investigating evidence for biomarkers for programmed cell death in nonencysted cells. If so, then later studies need to determine the underlying algorithm as well as identifying participating molecules. Another important question is, what is 80% of the cell cycle? There is an excellent review by Byers et al. [22] on the cell cycle of* Acanthamoeba* and it is recommended for further reading. The cell cycle progression, cell cycle arrest, and encystment are highly complex and depend on variable environmental conditions, availability of nutrients, and amoebae populations. For simplicity, we can conclude that asynchronous cultures of* Acanthamoeba* show a lack of the G1 phase (presynthetic gap), 2-3% S phase (synthesis), 85–90% G2 phase (postsynthetic gap), and 8–10% M phase (mitosis) [22]. This suggests that, for asynchronous cultures, nutrient deprivation triggers encystment in cells in the G2 phase. Does this mean that the programmed cell death occurs during the remaining phases? Notably, G2 phase occupies up to 90% of the cell cycle leaving behind a very limited window in which to execute programmed cell death. Previously, Byers et al. [22] questioned whether encystment in* Acanthamoeba* occurs during a portion of the G2 phase. If so, then what are the subphases of G2? Is it possible that one of the subphases is responsible for encystment, while another subphase is responsible for the programmed cell death? Unfortunately, there is no available data but further study should explore the possibility of coexistence of encystment and programmed cell death during the G2 phase. In this context, cellular differentiation in* Acanthamoeba* is distinct from metazoans. In mammals, when a cell undergoes differentiation, it exits from the G1 phase of the cell cycle to enter into a quiescent state referred to as G0 [23], while apoptosis can occur during both the G1 and the G2 phase of the cell cycle, preceded by the cell growth arrest [24, 25].

**Figure 1:**
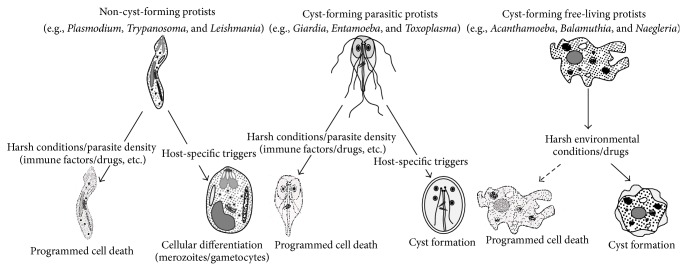
Stress management in parasitic and free-living protists.

An alternative explanation of coexistence of encystment and the programmed cell death is that the basis of functionally exclusive phenotypes (encystment* versus* programmed cell death) is subject to the trigger source or the type of trigger. For example, in the natural environment, encystment in this group is almost always stimulated by signals from extracellular sources (extrinsic signals) such as nutrient deprivation and extremes in pH, temperatures, and osmolarity. But how does this group of organisms respond to signals from intracellular sources (mitochondrial dysregulation/genomic instability/bacterial or viral infections)? [26, 27]. In their natural environment, is it plausible that cyst-forming free-living protists undergo encystment in response to extrinsic stress signals, while they execute programmed cell death in response to intrinsic stress signals? This would suggest that extracellular stress signals are channeled through the in-house stress management system, that is, encystment, while mitochondrial dysregulation/DNA damage would induce programmed cell death. The use of molecules such as hydrogen peroxide that are known to induce programmed cell death in several protists, as well as ultraviolet irradiation, would be useful to help elucidate both pathways. Given a repertoire of responses by cyst-forming free-living protists, it is challenging to study and correlate specific triggers, mediators, and executors with a specific phenotype. For example, various stress signals in* Acanthamoeba* induce distinct phenotypic responses such as growth inhibition and pseudocyst formation (bistability between trophozoite and cyst stage), may induce cyst formation, or may cause apoptosis, autophagy, or necrotic death [18, 19, 28–30]. In addition to the aforementioned,* Acanthamoeba* is known as the Trojan horse of the microbial world and feeds on endosymbiotic bacteria [3]. Future studies are needed to determine the possible role of amoebal endosymbionts in amoebal cell death or cyst formation. Overall, unlike non-cyst-forming protists, this group of organisms is adept in responding to variations in environmental conditions (extrinsic signals) as well as intracellular dysfunction (Figure 1). The coexistence of both encystment and the programmed cell death functions in cyst-forming free-living protists is a fascinating area of research. In this context, organisms such as* Acanthamoeba* can serve as an excellent model to study cellular differentiation process and cellular death akin to programmed cell death. A complete understanding of the fundamental principles of genome evolution and biochemical pathways of cellular differentiation and death offers unprecedented opportunities to counter detrimental outcomes.

## 5. Programmed Cell Death in Cyst-Forming Parasitic Protists 

Cyst formation is an integral part of the life cycle of cyst-forming parasitic protists (examples are* Toxoplasma gondii*,* Giardia lamblia*,* Entamoeba histolytica*,* Cryptosporidium parvum*, and* Balantidium coli*) and occurs inside the parasitized host. The encysted form leaves the host in search of a new host. In the next host, the encysted form excysts, multiplies, and establishes an infection and then forms cysts once again to complete the life cycle. They are unable to propagate independently in the environment as they remain encysted (dormant). They only come to life (the vegetative form) inside the parasitized host. Hence they are different from cyst-forming free-living protists, which can maintain an independent life in the environment. The key objective of this group of parasitic protists (obligate parasites) is to infect new hosts and expand in numbers for their species survival/dominance. Despite the fundamental difference between cyst-forming parasitic protists and cyst-forming free-living protists of dependency and independency of a host, respectively, cyst formation is a requirement to survive environmental stresses during the transmission of both parasites. Except for conceptual resemblance in encystment between cyst-forming parasitic protists and cyst-forming free-living protists, it is envisaged that there must be fundamental differences in triggers, mediators, and molecular events of encystment in protists that encyst inside a vertebrate host versus protists that normally encyst in the environment. Cyst formation in free-living protists is a response to harsh environmental conditions, while cyst formation in parasitic protists requires host-specific triggers. For example, neither* G. lamblia* nor* E. histolytica*/*C. parvum* encyst in response to typical harsh environmental conditions such as nutrient deprivation and extremes in pH and temperatures [31, 32]. Naturally, the question arises: how do they respond to the harsh environmental conditions? As per our assumption, protists that need to parasitize a host to complete their life cycle and are unable to live independently must have in-built system(s) to respond to harsh environmental conditions, possibly via death akin to programmed cell death. In support, several cyst-forming parasitic protists have been shown to undergo cell death including* E. histolytica* and* G. lamblia* [33–36]. In addition,* Entamoeba* does not have mitochondria triggering intrinsic cell death such as apoptosis, implying unusual cell death mechanisms. Overall, the available evidence suggests that both encystment and the cell death functions coexist in cyst-forming parasitic protists but the precise triggers, modulators, and executors are incompletely understood.

## 6. Conclusions

There is a long way to go to compute mechanics of programmed cell death in protists. From the currently available evidence, it is reasonable to suggest that protists that are dependent on a host to complete their life cycle (non-cyst-forming and cyst-forming parasitic protists, i.e., true/obligate parasites) execute programmed cell death or modulate host cell death. For example, parasitic protists like microsporidia have developed a strategy that inhibit programmed cell death of the host cell by preventing normal host cell division, thereby averting premature exposure of the developing stages of the parasite to the environment (extracellular or external), a potentially catastrophic event for the parasite.* Encephalitozoon* and* Nosema algerae* [15] or* Toxoplasma gondii* [14] appears to impart resistance to apoptotic signals within the host cell. It is therefore logical to assume that it is in the parasite's interest to cause an arrest in the host cell cycle, thereby producing a stable niche for itself.

In contrast, death akin to programmed cell death in cyst-forming free-living protists (facultative parasites) that do not require a host to complete their life cycle is unclear. The coexistence of programmed cell death and encystment is an important area of study to understand distinct cellular differentiation processes. In addition to evolutionary perspectives, these investigations will yield information of specific molecules/pathways that are missing in mammals to induce pathogen death for targeted therapy.

## Key Findings

- Protists dependent on a host to complete their life cycle execute programmed cell death.
- In contrast, programmed cell death in cyst-forming free-living protists independent of a host to complete their life cycle is unclear.
- Coexistence of PCD and encystment will help understand distinct cellular differentiation processes.
- Findings will identify specific molecules/pathways that are missing in mammals to induce pathogen death for targeted therapy.
